# Statins Reduce Melanoma Development and Metastasis through MICA Overexpression

**DOI:** 10.3389/fimmu.2013.00062

**Published:** 2013-03-13

**Authors:** Christine Pich, Iotefa Teiti, Philippe Rochaix, Bernard Mariamé, Bettina Couderc, Gilles Favre, Anne-Françoise Tilkin-Mariamé

**Affiliations:** ^1^INSERM/UPS UMR 1037, Cancer Research Center of ToulouseToulouse, France; ^2^Institut Claudius RegaudToulouse, France; ^3^Université Paul SabatierToulouse, France; ^4^INSERM/UPS UMR 1043, Physiopathology Center of Toulouse-PurpanToulouse, France; ^5^EA 4553, University Cancer ClinicToulouse, France

**Keywords:** statins, melanoma, MICA, NK cells

## Abstract

Survival of melanoma patients after metastases detection remains short. Several clinical trials have shown moderate efficiency in improving patient survival, and the search for pharmacological agents to enhance the immune response and reduce melanoma metastases is still necessary. Statins block the mevalonate pathway, which leads to decreases in GTPase isoprenylation and activity, particularly those of the Ras superfamily. They are widely used as hypocholesterolemic agents in cardiovascular diseases and several studies have shown that they also have protective effects against cancers. Furthermore, we have previously demonstrated that treatment of melanoma cells with inhibitors of the mevalonate pathway, such as statins, favor the development of specific adaptive immune responses against these tumors. In the present study, we tested statin impact on the innate immune response against human metastatic melanoma cells. Our data shows that treatment of two human melanoma cell lines with statins induced a weak but significant increase of MHC class I Chain-related protein A (MICA) membrane expression. Peroxisome Proliferator-Activated Receptor gamma is involved in this statin-induced MICA overexpression, which is independent of Ras and Rho GTPase signaling pathways. Interestingly, this MICA overexpression makes melanoma cells more sensitive to *in vitro* lysis by NK cells. The impact of statin treatment on *in vivo* development of melanoma tumors and metastases was investigated in nude mice, because murine NK cells, which express NKG2D receptors, are able to recognize and kill human tumor cells expressing MICA. The results demonstrated that both local tumor growth and pulmonary metastases were strongly inhibited in nude mice injected with statin-treated melanoma cells. These results suggest that statins could be effective in melanoma immunotherapy treatments.

## Introduction

Cutaneous melanomas are the most aggressive skin cancers. They arise from melanocytes and, after horizontal and vertical growth, extravasate into the draining lymph nodes and blood vessels, from which they can metastasize. Patient survival following metastatic detection is usually short. Therefore, current treatments target metastatic growth. Inhibitors of BRAF mutated V600E were validated by several clinical trials but their efficiency is not long lasting and is mostly restricted to BRAF V600E mutated tumors (Flaherty et al., 2010; Weeraratna, 2012). Other treatments with antibodies directed against the CTLA-4 molecule have shown efficiency in metastatic melanoma (Hodi et al., 2010), but these antibodies can also induce deleterious inflammation (Berman et al., 2010). Thus, the search for pharmacological agents to enhance the immune response to reduce melanoma metastases is still necessary.

Statins are used by millions of people as hypocholesterolemic agents in cardiovascular and cerebrovascular diseases. Additionally, retrospective studies have shown that, besides this hypocholesterolemic activity, statins can also have a protective effect against the development of cancers, particularly melanomas (Boudreau et al., 2010; Jacobs et al., 2011). Statins are HMG-CoA reductase inhibitors, which block the mevalonate synthesis pathway. By inhibiting this metabolic pathway, statins also inhibit the activity of numerous GTPases of the Ras superfamily as they require the isoprenoid chains, synthesized from the mevalonate, for their function and localization (Rikitake and Hirata, 2009). Statins also have pleiotropic effects, including the enhancement of Peroxisome Proliferator-Activated Receptor gamma (PPARγ) activities (Balakumar and Mahadevan, 2012). PPARγ is a transcription factor that is overexpressed in several types of cancer (Peters et al., 2012), particularly melanomas (Meyer et al., 2009).

We have previously shown that treatments of melanoma cells with mevalonate pathway pharmacological inhibitors, notably statins, and in combination with IFNγ favor the anti-tumor adaptive immune response by inducing MHC class I and costimulatory molecule (CD80/CD86) overexpression (Tilkin-Mariame et al., 2005; Sarrabayrouse et al., 2010). We then asked the question whether or not these inhibitors could also favor the anti-melanoma innate immune response. We were particularly interested in NK cells, which are among the most efficient effectors of the innate immune system and also recognize several molecules expressed on the surface of both stressed and tumor cells. A critical receptor expressed on NK cells is the NKG2D activating receptor, which is able to recognize tumor cells from murine or human origin (Cerwenka and Lanier, 2001; Fuertes et al., 2008). On tumor or stressed cells, this type II transmembrane receptor recognizes ligands, such as UL-Binding Proteins (ULBPs) and MHC class I Chain-related protein A (MICA) and B (MICB), all related to the MHC class I molecules (Lanier, 2008). NKG2D expression is essential for NK cell control of tumor development, as illustrated by NKG2D-KO mice, which develop numerous tumors and die prematurely (Guerra et al., 2008).

In the present study, we analyzed statins’ ability to regulate MICA expression in human melanoma cells and the consequences of this treatment on melanoma development. We demonstrated that treatment of two human melanoma cells with atorvastatin or lovastatin induced a weak but reproducible MICA membrane overexpression, provoking an increase of melanoma cell sensitivity to NK cell lysis *in vitro*. Moreover, we observed *in vivo* that atorvastatin pretreatment of these melanoma cells strongly reduced both local melanoma growth following subcutaneous injection as well as pulmonary metastases implantation after intravenous injections. This data suggests that statins could be interesting pharmacological inhibitors for melanoma immunotherapy as they favor the innate immune response against tumor cells.

## Results

### Statin treatment induces MICA overexpression and increases melanoma sensitivity to NK-killing

**Figure 1:**
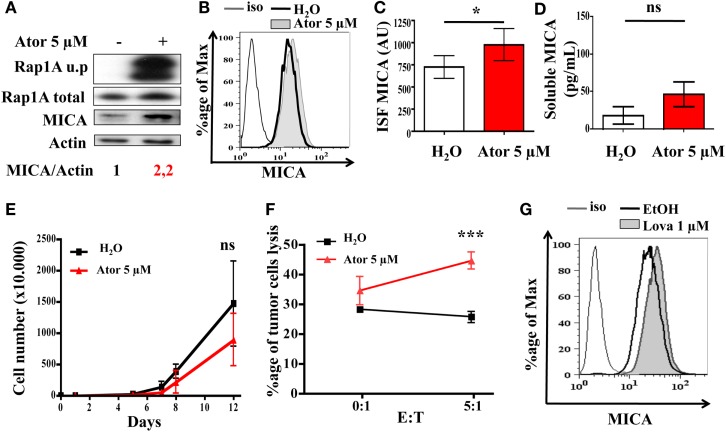
**Statin treatment induces MICA overexpression and increases NK-dependent cytotoxicity**. LB1319-MEL cells were treated with 5 μM atorvastatin for 48 h (Ator) or untreated (H_2_O). The atorvastatin treatment efficiency was controlled by the analysis of Rap1A unprenylation (Rap1A u.p) compared to the total protein (Rap1A total) **(A)**. MICA total expression was analyzed by western blot **(A)**, membrane expression by flow cytometry **(B,C)**, and cleavage by ELISA **(D)** (*n* = 3). To evaluate membrane antigen expression, the index of specific fluorescence (ISF) was calculated as indicated in the Section “Materials and Methods” **(C)**. Results are expressed as mean ± SD [error bars, *n* = 5 experiments **(C)** or 3 experiments **(D)**]. *P < 0.5; versus control condition using the Student *t*-test. Every 2–3 days pretreated (Ator) and untreated (H_2_O) LB1319-MEL cells were counted to evaluate the *in vitro* proliferation **(E)**. ns; versus control condition using the two-way ANOVA test. Percentage of lysed LB1319-MEL cells pretreated (Ator) or untreated (H_2_O) after coculture with murine NK cells was evaluated with Cytoxilux kit (Onco-immumin, Interchim). Murine NK cells were first isolated from three C57BL/6 spleens with CD49b MicroBeads (Miltenyi Biotec) **(F)**. ***P < 0.001; versus control condition using the two-way ANOVA test. LB1319-MEL cells were treated with 1 μM lovastatin for 48 h, and MICA membrane expression was analyzed by flow cytometry **(G)**.

**Figure A1:**
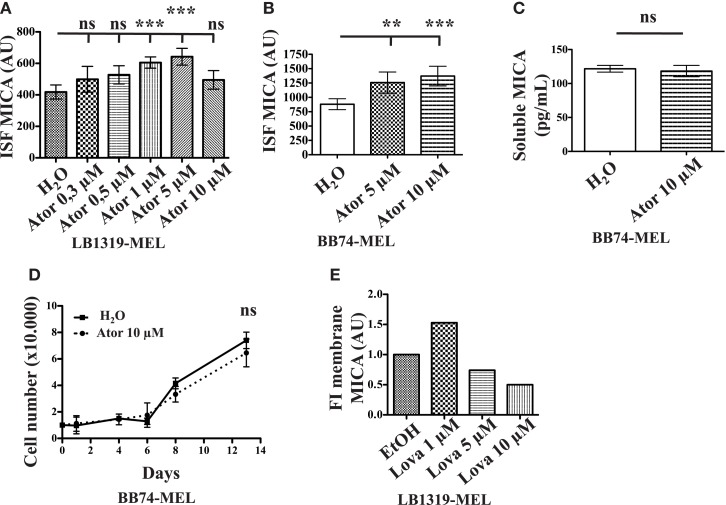
**Atorvastatin and lovastatin treatments of LB1319-MEL and BB74-MEL cells induce MICA membrane overexpression**. LB1319-MEL cells were treated with increasing concentrations of atorvastatin and membrane MICA expression was analyzed by flow cytometry **(A)**. BB74-MEL cells were treated with 5 or 10 μM atorvastatin for 48 h (Ator) or untreated (H_2_O). MICA membrane expression was analyzed by flow cytometry **(B)** and cleavage by ELISA **(C)**. **P < 0.01; ***P < 0.001; versus control condition using the Tukey ANOVA test. Every 2–3 days pretreated (Ator) and untreated (H_2_O) BB74-MEL cells were counted to evaluate the *in vitro* proliferation **(D)**. ns; versus control condition using the two-way ANOVA test. LB1319-MEL cells were treated with increasing concentrations of lovastatin and membrane MICA expression was analyzed **(E)**.

We tested whether treatment of human melanoma cells with statins could render these cells more immunogenic and more sensitive to NK cell destruction. LB1319-MEL human melanoma cells were treated for 48 h with 5 μM atorvastatin. This treatment induced a 2.2-fold total MICA protein enhancement (Figure 1A) and also weakly enhanced MICA membrane expression (Figures 1B,C), but not the deleterious cleavage of membrane expressed MICA (Figure 1D). The 5 μM dose of atorvastatin for 48 h was chosen after dose-response experiments as illustrated in Figure A1A in Appendix. This atorvastatin treatment is not toxic to LB1319-MEL cells, as their *in vitro* proliferation rate following treatment is still similar to the control (Figure 1E). Similar results were obtained with another human melanoma cell line, BB74-MEL, which was treated with atorvastatin at 5 or 10 μM. Similar to LB1319-MEL cells, the BB74-MEL cells also exhibited weak membrane MICA overexpression in response to statins (Figure A1B in Appendix) without increasing the cleavage of membrane expressed MICA (Figure A1C in Appendix) or toxicity (Figure A1D in Appendix).

Recent studies showed that the increase of MICA and MICB expression on target tumor cells induced an increase in sensitivity to lysis by NK cells (Zhang et al., 2009; Chavez-Blanco et al., 2011). We therefore tested whether or not atorvastatin plays a role in increasing the sensitivity of melanoma cells to NK cell-mediated death. We isolated fresh NK cells from C57BL/6 splenocytes using magnetic cell separation (Miltenyi Biotec) and cocultivated them for 1 h with LB1319-MEL target cells either untreated or pretreated with atorvastatin. FACS analysis of tumor cell lysis showed that atorvastatin treatment induced a twofold increase in LB1319-MEL cell death as compared to untreated target cells (Figure 1F). In this experiment, corresponding to previously shown data, murine NK cells were able to recognize and kill human target cells by interaction of their NKG2D receptors with specific ligands expressed on human target cells, such as MICA, MICB, and ULBP (Cerwenka and Lanier, 2001; Fuertes et al., 2008).

To test whether another statin could also induce MICA membrane overexpression, LB1319-MEL cells were treated with 1 μM lovastatin for 48 h and MICA expression was analyzed by flow cytometry. The 1 μM dose of lovastatin was chosen after dose-response experiments as illustrated in Figure A1E in Appendix. Similarly to atorvastatin, lovastatin induced a weak MICA membrane overexpression (Figure 1G).

Altogether, this data shows that statin treatment of human melanoma cells enhanced MICA protein and membrane expression and that these treatments were not toxic, but increased the sensitivity of the tumor cells to NK-induced cell death *in vitro*.

### Atorvastatin treatment of LB1319-MEL cells slows tumor growth and reduces metastatic pulmonary implantation

**Figure 2:**
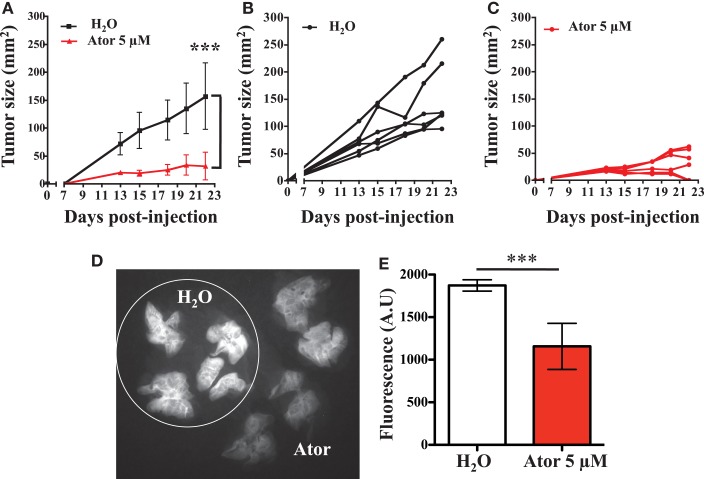
**Atorvastatin treatment inhibits melanoma *in vivo* development and metastasis**. LB1319-MEL cells untreated (H_2_O) or pretreated with atorvastatin 5 μM for 48 h (Ator) were injected subcutaneously into the flank of six nude mice. Tumor growth was measured every 2–3 days **(A)**. ***P < 0.001; versus control condition using the two-way ANOVA test. Tumor growths for each mice are represented **(B,C)**. LB1319-MEL cells untreated (H_2_O) or pretreated with 5 μM atorvastatin for 48 h (Ator) were injected intravenously. Fifteen days later IntegriSense was intravenously injected and the mice were sacrificed 30 h later. The lungs appeared fluorescent and the IntegriSense fluorescent signal was analyzed by Fluobeam^®^ camera. The image is representative of one experiment **(D)**. The fluorescent signal was quantified **(E)**. ***P < 0.001; versus control condition using the Student *t*-test.

**Figure A2:**
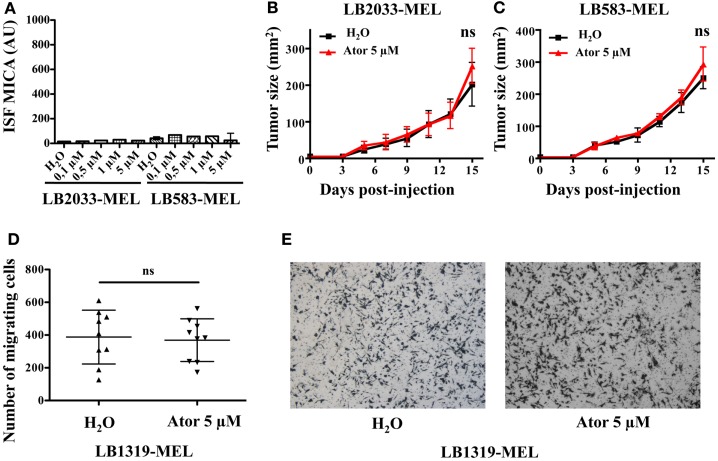
**Atorvastatin treatment had no effect on MICA negative melanoma cell lines *in vivo* tumor growth and on *in vitro* tumor migration**. LB2033-MEL and LB583-MEL cells were treated with increasing concentrations of atorvastatin for 48 h and MICA membrane expression was analyzed by flow cytometry **(A)**. ns; versus control condition using the Tukey ANOVA test. LB2033-MEL and LB583-MEL cells untreated (H_2_O) or pretreated with atorvastatin 5 μM for 48 h (Ator) were injected subcutaneously into the flank of nude mice. Tumor growth was measured every 2–3 days **(B,C)**. ns; versus control condition using the two-way ANOVA test. *In vitro* migration of LB1319-MEL cells untreated (H_2_O) or pretreated with atorvastatin 5 μM for 48 h (Ator) was analyzed using transwell assays **(D)**. ns; versus control condition using the Student *t*-test. Images are representative of the migrating cells **(E)**.

Based on our *in vitro* conclusions, we wondered if atorvastatin treatment of melanoma cells could increase sensitivity to the anti-tumor innate immune response in a mouse model. In summary, we performed subcutaneous injections of LB1319-MEL cells, either untreated or pretreated with 5 μM atorvastatin for 48 h, into the flank of nude mice. We were not able to perform this assay with BB74-MEL cells, as they do not grow in the nude mice. The resulting tumors showed that LB1319-MEL cells pretreated with atorvastatin grew drastically (*p* < 0.005) slower than untreated cells (Figure 2A). Interestingly, two mice among the six injected with pretreated LB1319-MEL cells rejected their tumor (Figure 2C), whereas no rejection was observed among the mice injected with untreated LB1319-MEL cells (Figure 2B). These results are consistent with the statin-induced MICA overexpression and enhancement of *in vitro* tumor cell death by NK cells. Furthermore, we used two other human melanoma cell lines: LB2033-MEL and LB583-MEL. These cells are MICA negative and remained negative following atorvastatin treatment as illustrated in Figure A2A in Appendix. We injected subcutaneously these melanoma cells either untreated or pretreated, as LB1319-MEL, with 5 μM atorvastatin. The resulting tumors showed that negative MICA cells both pretreated and untreated grew similarly (Figures A2B,C in Appendix). These results confirmed the involvement of statin-induced MICA overexpression in the tumor growth.

We also tested the effect of atorvastatin treatment on the *in vitro* migration of the LB1319-MEL cells. We did not observed any significant difference between the migration of treated and untreated cells (Figures A2D,E in Appendix). This data suggested that the atorvastatin-induced slow-down of *in vivo* tumor growth is not linked to an inhibition of tumor cells migration.

As NK cells play an important role in the control of metastatic processes (Mehlen and Puisieux, 2006), we assessed whether statin-induced MICA overexpression and subsequent sensitivity to the innate immune response could reduce the metastatic processes, more specifically the final step of pulmonary implantation. LB1319-MEL cells either untreated or pretreated with 5 μM atorvastatin were injected intravenously into the tail vein of nude mice and the animals were sacrificed 16 days later. Thirty hours before sacrifice, the tumor and neoangiogenesis sensor IntegriSense was injected intravenously (Kimura et al., 2009; Mery et al., 2011; He et al., 2012; Valdivia et al., 2012). This fluorescent reagent allowed visualization and quantification of tumor implantations and neoangiogenesis in the lungs (Figures 2D,E). We observed that untreated LB1319-MEL cells colonized the lungs and induced a considerable neoangiogenesis, while atorvastatin-treated LB1319-MEL cells were less invasive with strongly decreased neoangiogenesis.

These results showed that statin pretreatment of melanoma cells reduced *in vivo* tumor development and metastasis.

### Inhibition of GTPases of Ras superfamily does not induce MICA overexpression

Proteins of the Ras superfamily play regulatory roles on essential cellular functions (Heasman and Ridley, 2008). Their activity can be blocked by statin treatment, because statins block mevalonate production by HMG-CoA reductase and consequently inhibit the production of isoprenyl PyroPhosphates (PP), geranyl PP, and farnesyl PP, which are the precursor molecules necessary to produce isoprenoid chains. These isoprenoid chains bind to the carboxy-terminal end of Ras superfamily proteins and allow adequate cellular localization and activity. To explain why statin treatment induced MICA overexpression, we hypothesized that statin could block a negative regulator of MICA expression. These negative regulatory proteins, blocked by statin treatment, could be isoprenylated proteins of the Ras superfamily.

**Figure 3:**
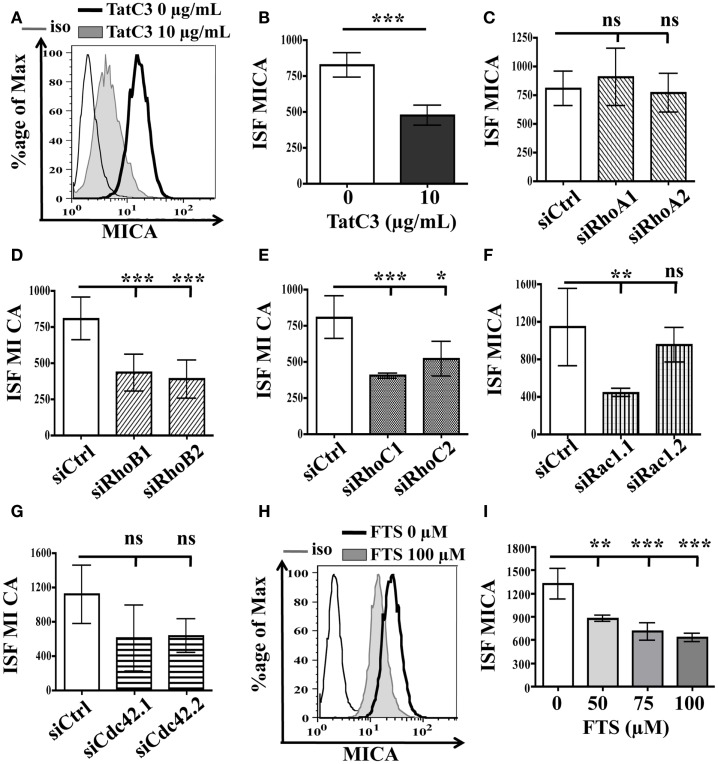
**Inhibition of Rho and Ras GTPases proteins do not induce MICA membrane overexpression**. LB1319-MEL cells were untreated or treated with TatC3 exoenzyme (TatC3) 10 μg/mL for 24 h **(A,B)**. LB1319-MEL cells were transfected with control siRNA (siCtrl) **(C–G)**, two RhoA-specific siRNAs (siRhoA1, siRhoA2) **(C)**, two RhoB-specific siRNAs (siRhoB1, siRhoB2) **(D)**, two RhoC-specific siRNAs (siRhoC1, siRhoC2) **(E)**, two Rac1-specific siRNAs (siRac1.1, siRac1.2) **(F)**, or two Cdc42-specific siRNAs (siCdc42.1, siCdc42.2) **(G)**. LB1319-MEL cells were treated or not with a Ras inhibitor, FTS, 50–100 μM for 24 h **(H,I)**. MICA membrane expression was analyzed by flow cytometry 72 h after transfection or 24 h after treatment. Representative illustrations of untreated cells (black) and of treated cells (filled in gray) with Tat-C3 exoenzyme **(A)** or FTS **(H)** are shown. To evaluate membrane antigen expression, the index of specific fluorescence (ISF) was calculated as indicated in the Section “Materials and Methods.” Results are expressed as mean ± SD (error bars, *n* = 3 experiments). *P < 0.5; **P < 0.01; ***P < 0.001 versus control condition using the Student *t*-test **(B)** or the Tukey ANOVA test **(C–I)**.

**Figure A3:**
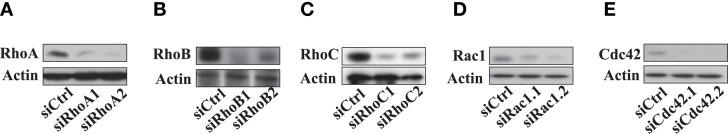
**Decrease of Rho proteins expression in LB1319-MEL cells transfected with Rho specific siRNAs**. The efficiency in decreasing Rho protein expression of Rho specific siRNAs transfections were analyzed by western blot, and actin was used as a control of a uniform loading. RhoA, RhoB, RhoC, Rac1, and Cdc42 expressions were analyzed to test their respective specific siRNAs efficiencies **(A–E)**.

We first tested the involvement of proteins of the Rho GTPases subfamily. The three highly related Rho isoforms, RhoA, B, and C share some effector proteins, but also show clear functional differences (Heasman and Ridley, 2008). To determine whether these Rho GTPases are involved in the regulation of MICA membrane expression, we used a bacterial inhibitor of RhoA, B, and C (Tat-C3 exoenzyme), which inhibits the Rho proteins activity by inducing ADP-ribosylation of these three GTPases. The results showed that treatment of LB1319-MEL cells with Tat-C3 actually induced downregulation of MICA rather than the expected increase (Figures 3A,B). Therefore, these Rho proteins effectively regulate MICA expression but in a positive way. We then used a siRNA strategy that allows the specific inhibition of RhoA, RhoB, or RhoC, to test whether one of them could behave differently and negatively regulate MICA expression. We first verified that these siRNAs were efficient in reducing RhoA, RhoB, or RhoC protein expression (Figures A3A–C in Appendix) and then tested their impact on MICA expression. Transfection of two RhoA-specific siRNAs in LB1319-MEL cells did not induce any modification of MICA expression (Figure 3C), while cells transfected with RhoB- or RhoC-siRNAs significantly reduced MICA expression as compared to siCtrl (Figures 3D,E). Consequently, these Rho proteins cannot be responsible for the increased expression of MICA induced by statin treatment.

We then tested the impact of siRNA-induced reduction of Rac1 and Cdc42, two other essential members of the Rho family. These proteins are known to be involved in the regulation of several cellular functions, including migration and gene transcription (Heasman and Ridley, 2008). SiRNA transfections successfully inhibited Rac1 and Cdc42 expression (Figures A3D,E in Appendix). We observed that Cdc42 inhibition had no statistical effect on MICA expression, while Rac1 inhibition actually reduced MICA expression in LB1319-MEL cells (Figures 3F,G). Altogether, these results show that these Rho proteins are not responsible for the statin-induced overexpression of MICA.

Next, we investigated the role of Ras proteins in statin-induced MICA overexpression. Ras GTPase activities can be inhibited by statin treatment, because these proteins need to be farnesylated on their C-terminal end for membrane anchorage and activity. To determine whether Ras proteins are involved in MICA overexpression, we used a biochemical inhibitor of Ras protein activity (*S*-FarneylThioSialicylic acid, FTS). The results showed that inhibition of Ras protein activity by FTS (50–100 μM for 24 h) also induced a decrease in MICA expression (Figures 3H,I). Therefore, Ras proteins are not responsible for statin-induced MICA overexpression.

### PPARγ and SAPK/JNK involvement in statin-induced MICA overexpression

**Figure 4:**
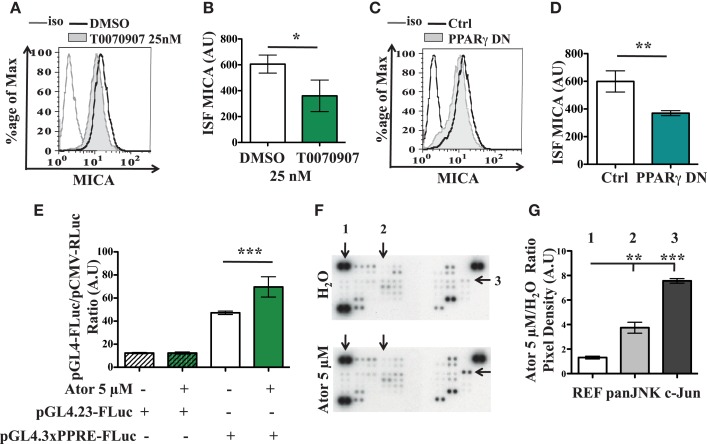
**PPARγ and SAPK/JNK are involved in atorvastatin-induced MICA overexpression**. LB1319-MEL cells were treated with DMSO or a PPARγ inhibitor, T0070907 25 nM for 24 h **(A,B)**. LB1319-MEL cells were transfected with an empty plasmid (Ctrl) or a plasmid encoding a PPARγ dominant negative mutant (PPARγ DN) **(C,D)**. MICA membrane expression was analyzed 24 h after treatment or transfection. Representative illustrations of controlled cells (black) and treated cells (filled in gray) with T0070907 **(A)** or PPARγ DN **(C)** are shown. The ISF were calculated **(B,D)**. LB1319-MEL cells were transfected with Firefly Luc control plasmids (pGL4.23-FLuc) or plasmid reporting the PPARγ PPRE activity (pGL4.3xPPRE-FLuc) and these cells were untreated (H_2_O) or treated with atorvastatin 5 μM (Ator 5 μM). Twenty-four hours later, luciferase assays were performed **(E)**. LB1319-MEL cells were untreated (H_2_O) or treated with atorvastatin 5 μM for 48 h and a Proteome Profiler Array was performed to compare kinases phosphorylation **(F,G)**. One, two, and three correspond respectively to a reference spot (REF), panJNK T182/Y185, T221/Y223 (panJNK), and c-Jun S63 (c-Jun) phosphorylations **(F)**. The densities of the signals were analyzed with ImageJ software and the ratios of atorvastatin on untreated conditions were calculated **(G)**. Results are expressed as mean ± SD (error bars, *n* = 3 experiments). *P < 0.5; **P < 0.01; ***P < 0.001 versus control condition using the Student *t*-test **(B,D)** or the Tukey ANOVA test **(E,G)**.

**Figure A4:**
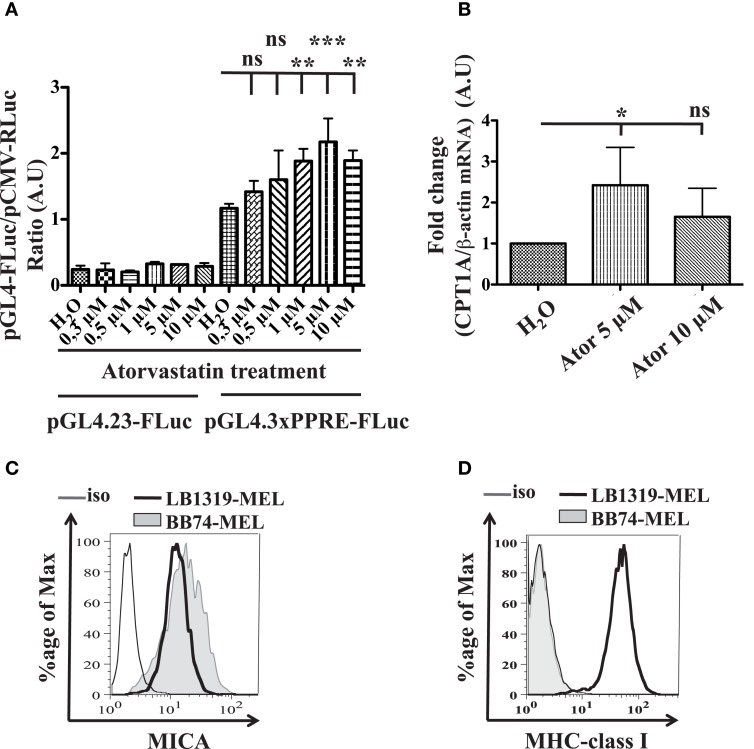
**Activation of PPARγ pathway by increasing doses of atorvastatin and MICA and MHC class I expression in LB1319-MEL versus BB47-MEL cell lines**. LB1319-MEL cells were transfected with Firefly Luc control plasmids (pGL4.23-FLuc) or plasmid reporting the PPARγ PPRE activity (pGL4.3xPPRE-FLuc) and these cells were untreated (H_2_O) or treated with increasing doses of atorvastatin from 0.3 to 10 μM. Twenty-four hours later, luciferase assays were performed **(A)**. LB1319-MEL cells were treated with 5 or 10 μM atorvastatin for 24 h or untreated (H_2_O) and CPT1A mRNA relative expression, normalized by β-actin, was detected by RT-qPCR **(B)**. Results are expressed as mean ± SD (error bars, *n* = 3 experiments). *P < 0.5; **P < 0.01; ***P < 0.001 versus control condition using the Tukey ANOVA test **(A,B)**. Expressions of MICA **(C)**, and MHC class I molecules **(D)** were compared between LB1319-MEL and BB74-MEL cell lines by flow cytometry.

As it is known that statins may affect the PPARγ pathway (Balakumar and Mahadevan, 2012), we checked the involvement of this factor in the statin-induced MICA overexpression. As shown in Figures 4A,B, treatment of the LB1319-MEL cells with T0070907, a specific inhibitor of the PPARγ pathway, led to a weak but significant and reproducible decrease of the membrane MICA expression. Additionally, transfection of an expression vector encoding a dominant negative mutant of PPARγ (PPARγ DN) had a similar effect (Figures 4C,D). Furthermore, LB1319-MEL cells transfected with a reporter vector containing three PPARγ Responsive Element (PPRE) cloned upstream of a luciferase gene (pGL4.3xPPRE-FLuc) exhibited a weak but significant increase of the luciferase expression following 5 μM atorvastatin treatment (Figure 4E). The treatment of LB1319-MEL cells with increasing doses of atorvastatin, from 0.3 to 10 μM, confirmed this data (Figure A4A in Appendix). Finally, CPT1A mRNAs were increased in LB1319-MEL cells following 5 μM atorvastatin treatment (Figure A4B in Appendix). This data confirm the involvement of PPARγ pathway since CPT1A is a target gene for PPARγ (Heinaniemi et al., 2007; Szatmari et al., 2007; Sharma et al., 2012). Therefore, it is possible that statins activate the PPARγ pathway, leading to downstream increases in MICA expression.

To assess the possible involvement of other pathways, we then performed a Human Phospho-Kinase Array (R&D systems). The obtained results, illustrated in Figures 4F,G, show that atorvastatin treatment enhanced phosphorylations of c-Jun N-terminal kinases (JKN, spot 2 in Figures 4F,G) and especially the c-Jun kinase on Serine 63 (c-Jun, spot 3 in Figures 4F,G). Atorvastatin could therefore increase MICA expression via the Stress Activated Protein Kinase/JNK (SAPK/JNK) pathway, as previously described in human kidney endothelial cells (Holmen et al., 2007).

Altogether, these results show that PPARγ and SAPK/JNK pathways are involved in the atorvastatin-induced MICA overexpression.

## Discussion

Melanomas are immunogenic tumors, which express tumor antigens and other molecules recognized by the effectors of innate and adaptive immune responses. The current metastatic melanoma immunotherapies have limited efficiency (Robert et al., 2011; Brahmer et al., 2012). Therefore, new molecules to enhance the immune response are still needed. Statins are well known for their hypocholesterolemic effect, but retrospective studies have shown that statins also have a protective effect against melanoma development (Jacobs et al., 2011). We previously showed that inhibitors of the mevalonate pathway, notably statins, favored an anti-melanoma adaptive immune response (Tilkin-Mariame et al., 2005; Sarrabayrouse et al., 2007, 2010). Here, we tested the ability of statins to enhance the NK-dependent innate immune response against human melanomas, particularly because these innate immune responses are essential for the control of metastases (Mehlen and Puisieux, 2006).

In two human melanoma cell lines, we showed that statin treatments were not toxic at the doses used and that they weakly and reproducibly upregulated MICA expression, which is a critical target for anti-tumor NK cells. Interestingly, this weak MICA overexpression was able to enhance melanoma cell sensitivity to NK cell lysis *in vitro*. Similarly, Zhang et al. (2009) showed that sodium butyrate treatment of HeLa and HepG2 cells induced a weak but significant MICA overexpression allowing NK cells induced-death enhancement. Furthermore as previously described using hydralazine and valproate treatment on epithelial cancer cell lines (Chavez-Blanco et al., 2011), our treatment of melanoma cells with statins induced MICA membrane overexpression without increasing the level of soluble MICA avoiding tumor-induced suppression of NK cytotoxicity against the tumor. Moreover, the efficacy of this statin-induced MICA overexpression was quite obvious in our *in vivo* experiments performed in nude mice. The innate immune response of nude mice could be tested against human melanoma cells because these mice have NK cells bearing NKG2D receptors, which are able to recognize human antigens, like MICA (Cerwenka and Lanier, 2001; Fuertes et al., 2008). *In vivo* experiments showed that melanoma tumor growth, induced by subcutaneous injections of statin-pretreated melanoma cells, was strongly decreased compared to the tumors induced by untreated melanoma cells. Furthermore two mice out of six completely rejected atorvastatin pretreated tumor cells. Most importantly, statin pretreatment also induced a significant decrease in melanoma metastatic lung implantation and a highly significant modification and decrease of the neoangiogenesis of these lungs. These modifications were detected by IntegriSense fluorescence, which recognize αvβ3 integrins overexpressed in the lungs during cancer cells colonization (Kimura et al., 2009; Mery et al., 2011; He et al., 2012; Valdivia et al., 2012). *In vitro* tumor migration is not affected by atorvastatin treatment (Figures A2D,E in Appendix) therefore NK-killing of MICA overexpressing melanoma cells seem to be determinant for the reduction of melanoma development *in vivo*, as suggested by the differences of tumor growth between MICA negative (LB583-MEL and LB2033-MEL), MICA-low (untreated LB1319-MEL), and MICA-higher (atorvastatin-treated LB1319-MEL) melanoma cells.

These *in vivo* experiments could not be reproduced with the BB74-MEL model because these cells did not form tumors in nude mice. Two hypotheses could explain these results. The first is that the *in vitro* proliferation rate of BB74-MEL cells (Figure A1D in Appendix) is too slow compared to LB1319-MEL cells (Figure 1E). As we followed the *in vivo* tumor growth of both cell lines for the same amount of time, the BB74-MEL cells may not have had enough time to form tumors. The second possible explanation is that BB74-MEL cells could be rejected at once by the NK cells of the nude mice, because BB74-MEL cells express more MICA than LB1319-MEL cells (Figure A4C in Appendix) and do not express MHC class I molecules, which inhibit NK cells activity (Figure A4D in Appendix).

In an effort to determine the mechanism of action of this statin-induced MICA overexpression, we came to the unexpected conclusion that it is not induced by members of the Ras superfamily. Experiments done with bacterial, biochemical inhibitors, or siRNAs specific for Ras and Rho GTPases could not phenocopy statin-induced MICA overexpression. However, two other statin-altered pathways (Holmen et al., 2007; Balakumar and Mahadevan, 2012) seem to be involved, namely PPARγ and SAPK/JNK. Inhibitors of PPARγ factor induced a weak downregulation of MICA expression, while treatment with atorvastatin increased the activity of a synthetic promoter containing PPREs. Similarly, atorvastatin treatment also resulted in increased phosphorylation of JNK and c-Jun. The respective roles of signaling pathways involving PPARγ and SAPK/JNK in melanoma development and in MICA expression are still unknown and should be analyzed in further studies.

In previous works, we showed that inhibitors of the Rho GTPases favored adaptive anti-melanoma immune response (Tilkin-Mariame et al., 2005; Sarrabayrouse et al., 2007, 2010). In the present paper, we demonstrate that statins have another beneficial role in modifying melanoma cells, which is to render them more sensitive to the NK-killing. These results reinforce the idea that statins could be effective drugs for an immuno-based therapy of melanoma, by favoring adaptive and innate responses as well as reducing the metastatic process.

## Materials and Methods

### Tumor cell lines and animals

The human melanoma cell lines LB1319-MEL, BB74-MEL, LB2033-MEL, and LB583-MEL were kindly provided by Pr. T. Boon (Ludwig Institute for Cancer Research, Brussels). They were maintained in culture by serial passages in culture medium composed of RPMI 1640 medium (Lonza) supplemented with 10% FCS, 1 mM glutamine, 1% penicillin-streptomycin-amphotericinB (Lonza), and they were monthly tested to be mycoplasma-free. Six- to nine-week-old female NMRI nude mice were obtained from Elevages Janvier. The experiments in mice have been done in the appropriate conditions of husbandry, experimentation and care, controlled by the Ethic Comity of the Institut Claudius Regaud under the control of the Regional Comity of Midi-Pyrénées (France). Our protocols were validated and received the agreement number ICR-2009-0011.

### Treatment of melanoma cells

Melanoma cells were treated with a synthetic statin: atorvastatin (Pfizer) at different doses from 0.3 to 10 μM for 24 or 48 h, or a natural statin: lovastatin (Calbiochem) at 1, 5, or 10 μM for 48 h, or a RhoA, B, and C inhibitor: Tat-C3 exoenzyme (produced in our laboratory) (Sarrabayrouse et al., 2007) at 10 μg/mL for 24 h, or with a Ras inhibitor: *S*-FarneylThioSialicylic acid (Interchim) at increasing concentrations from 50 to 100 μM for 24 h, or a PPARγ inhibitor: T0070907 (Calbiochem) at 25 nM for 24 h. Untreated cells correspond to cells treated with equivalent volume of H_2_O for atorvastatin, Ethanol (EtOH) for lovastatin, NaCl for Tat-C3 exoenzyme, DMSO for FTS and T0070907.

### Transfection of siRNAs or plasmids

Melanoma cells were transiently transfected with siRNAs as previously described (Sarrabayrouse et al., 2007). Briefly, 5 × 10^5^ cells were transfected with 20 nM of siRNAs using Oligofectamine (Invitrogen). The siRNA duplexes were purchased from Eurogentec: non-targeting siControl (siCtrl; GACGUGGGACUGAAGGGGU-TT), siRhoA1 (GAAGUCAAGCAUUUCUGUC-TT), siRhoA2 (GCAGGUAGAGUUGGCUUUG-TT), siRhoB1 (CUAUGUGGGCCGACAUUGAG-TT), siRhoB2 (CCGUCUUCGAGAACUAUGU-TT), siRhoC1 (UAAGAAGGACCUGAGGCAA-TT), siRhoC2 (GACUAUGAUCGACUGCGC-TT), siRac1.1 (CACCACUGUCCCAACACUC-TT), siRac1.2 (AAGGAGAUUGGUGCUGUAA-TT), siCdc42.1 (GAUAACUCACCACUGUCCA-TT), siCdc42.2 (GACUCCUUUCUUGCUUGUU-TT).

Melanoma cells were transiently transfected with a plasmid encoding PPARγ dominant negative proteins. Briefly, 5 × 10^5^ cells were transfected with 500 ng of plasmids with JetPrime (Invitrogen) according to the manufacturer’s instructions.

### Flow cytometry analyses

Melanoma cells (1 × 10^6^) were stained with PE-conjugated anti-MICA mAb and isotype control purchased from R&D Systems. After 1 h incubation the stained cells were analyzed on a FACSCalibur (Becton Dickinson) and the results were analyzed with FlowJo software (Tree Star). To evaluate membrane antigen expression on several independent experiments, the index of specific fluorescence (ISF) was determined. This was calculated using the following formula:

(1) $$\frac{\begin{array}{c} (\text{Median Fluorescence Intensity MFI with specific antibody}\\ -\text{MFI with isotype control})\times 100 \end{array}}{\text{MFI with isotype control}}$$

### Western blot analyses

Cells were lysed in lysis buffer (20 mM Tris pH 7.6, 150 mM NaCl, 2 mM EDTA, 0.1% SDS, 0.5% NP-40, 1% proteases, and phosphatases inhibitors cocktails) and protein extracts were prepared by the standard procedure and then separated (50–200 μg protein/lane) on SDS-PAGE gels. Proteins were blotted onto polyvinylidendifluoride membranes. The filters were incubated at 4°C overnight with primary antibodies against MICA (R&D Systems). Actin was used as a loading control (Chemicon). The membranes (Hybond-p, Amersham Biosciences) were then incubated with HRP-labeled secondary antibody (Immunotech) for 1 h at room temperature (RT) and then detected by chemiluminescence detection kit (ECL, Pierce). Band intensities were quantified using ImageJ software (National Institute of Health).

### ELISA assay

ELISA assays were performed using MICA DuoSet ELISA (R&D Systems). Briefly, capture antibodies were coated onto 96-wells plates overnight. Wells were washed and incubated with blocking reagent for 1 h at RT. One hundred microliters of the supernatants to be tested were added in triplicate for 2 h and detection antibody (in 2% goat serum; Lonza) was next incubated for 2 h at RT. One hundred microliters of Streptavidin-HRP were incubated for 20 min in the dark at RT and the reaction was revealed by TBM (Thermo-Scientific) after 20 min in the dark at RT. The reaction was stopped by addition of sulfuric acid 1 N. Optical density was measured on a Labtech spectrophotometer at 450 nm.

### NK cells isolation

NK cells isolations were performed using CD49b (DX5) MicroBeads (Miltenyi Biotec). Briefly, lymphocytes were isolated from three C57BL/6 spleens. Lymphocytes were then labeled with CD49b antibody coated with magnetic MicroBeads and applied onto a column placed on a magnetic support. The column was washed and unlabeled cells were collected. The CD49b-labeled NK cells were collected with PBS after the column removal from the magnetic support. NK cells purification was evaluated using FITC-conjugated CD49b antibody (>90%).

### Analysis of NK cells cytotoxic activity

We used Cytoxilux kit (Onco-immumin, Interchim) to investigate NK cells cytotoxic activity by flow cytometry. Briefly 1 × 10^6^ LB1319-MEL cells pretreated either with H_2_O or 5 μM atorvastatin for 48 h were stained with targets marker TFL-4 (FL-4) for 20 min at 37°C and then washed with PBS. Stained target cells were then diluted at 8 × 10^4^ cells per 100 μL of culture medium. The effector NK cells (E) were incubated with the stained target cells (T) for 1 h at 37°C at 5:1 E/T ratio in 96-well plates (Dutscher) in triplicate. Supernatant of each well was then discarded and 75 μL of caspase substrate, which is FITC fluorescent when cleaved (FL-1) or 75 μL of Wash Buffer for control conditions were added. Cells were finally washed with Wash Buffer and resuspended in PBS for analysis on a FACSCalibur. The percentage of cytotoxicity was calculated as the percentage of dead target cells, stained both green (FL-1+) and blue (FL-4+), among the total number of target cells (all FL-4+ cells).

### Subcutaneous tumor growth

To study the tumor growth, NMRI nude mice were injected subcutaneously (s.c.) with 5 × 10^6^ LB1319-MEL cells or 2 × 10^6^ LB2033-MEL or LB583-MEL cells either untreated or pretreated with 5 μM atorvastatin for 48 h. The melanoma cells were washed thrice in PBS before the injections to completely eliminate the statin. Animals were monitored for tumor growth every 2–3 days by palpation and diameters of the tumors were measured using a Vernier caliper. Tumor-bearing animals were sacrificed at day 15 or 22 after tumor injection. At this time no mice were died and the tumors did not display severe ulceration or reached a size of 300 mm^2^. For the mice that had rejected the tumor cells, experiments were terminated 9 weeks after tumor implantation. Two groups of three mice were tested and the experiments were done twice. Results are expressed as surface ±SD (error bars, *n* = 6 mice). Statistical analysis was performed using a two-way ANOVA test.

### Pulmonary metastases implantation

To study pulmonary metastases implantation, NMRI nude mice were injected i.v. with 1 × 10^6^ LB1319-MEL cells either untreated or pretreated 48 h with 5 μM atorvastatin. The melanoma cells were washed thrice in PBS before the injections to completely eliminate the statin. These i.v. injections in the tail vein of melanoma cells followed by lungs examination for tumor colonization allows controlled and quantitative experimentation of the last step of metastasis: the organ colonization. Mice were sacrificed 16 days later, at this time no mice were died. Mice were i.v. injected with 100 μL of IntegriSense (Perkin-Elmer) 30 h before sacrifice. The fluorescence in the lungs was analyzed by Fluobeam^®^ camera (690–700 nm) for 120 ms exposition and the fluorescent signals were quantified using ImageJ software (National Institute of Health). The experiments included three or four mice/group and were done twice.

### *In vitro* assays for cell migration

The migration assay was performed with 8-μm pore size transwell system (BD Biosciences). Cells (1 × 10^4^/well) were added with serum-free medium in the upper compartment of the filter. The bottom chamber was filled with complete medium. After 24 h, cells on the bottom surface of the filter were stained and counted under an Eclipse Ti microscope (Nikon Instruments; objective 20×) and a CoolSNAP HQ2 camera (Photometrics) in three randomized fields of 505 mm^2^.

### Luciferase assays

The reporter vector for PPARγ, pGL4.3xPPRE-FLuc plasmid containing PPRE sequences, was homemade constructed from the pB3xPPRE-Luc in an empty vector pGL4.23-FLuc (Rauwel et al., 2010) expressing Firefly Luciferase (FLuc). The pGL4.3xPPRE and the pGL4.23 were transfected using JetPrime (Invitrogen). The pCMV-RLuc plasmid expressing Renilla Luciferase (RLuc) was co-transfected as an internal control. Briefly, 3 × 10^4^ cells were transfected with the reporter vectors as described above. Four hours after, transfection was stopped by removing the medium and cells were treated with H_2_O or atorvastatin. Twenty-four hours later, luciferase activities were measured using the Dual Luciferase Assay System (Promega).

### Real-Time quantitative PCR

Total RNA of LB1319-MEL cells untreated and treated with atorvastatin 5 or 10 μM for 24 h were isolated using the RNeasy mini kit (Qiagen) combined with the RNase-Free DNase set (Qiagen) according to the manufacturer’s instructions, then reverse-transcribed using the iScript cDNA synthesis kit (BioRad). Quantitative real-time PCR was performed with a CFX96 Touch Real-Time PCR detection system (BioRad) using iQ SYBR Green Supermix (BioRad). We used β-actin as a housekeeping gene. Primers sequences were: CPT1A forward 5′TCG-ATT-TTC-AAG-GGT-CTT-CG3′, CPT1A reverse 5′CAC-AAC-GAT-CAG-CAA-ACT-GG3′, β-actin forward 5′TCC-CTG-GAG-AAG-AGC-TAC-GA3′, β-actin reverse 5′-AAG-GAA-GGC-TGG-AAG-AG3′. The relative fold change was calculated using the following formula: 2^−((Ct CPT1A − Ct β-actin) treated cells/(Ct CPT1A – Ct β-actin) untreated cells)^.

### Proteome profiler array

Proteome Profiler Arrays were performed using Human Phospho-Kinase Array Kit (R&D Systems). Briefly, 5 × 10^5^ LB1319-MEL cells, either untreated (H_2_O) or treated with 5 μM atorvastatin, were lysed in the Lysis buffer. Supernatants were incubated on the blocked membranes overnight at 4°C. Membranes were washed and incubated with detection antibodies cocktails for 2 h at RT. Membranes were washed and incubated with 1 mL of diluted Streptavidin-HRP for 30 min in the dark at RT. Then the phospho-kinases were detected using chemiluminescence detection. Spot intensities were quantified using ImageJ software (National Institute of Health).

## Conflict of Interest Statement

The authors declare that the research was conducted in the absence of any commercial or financial relationships that could be construed as a potential conflict of interest.
